# Supplementary material for: Comparative MD simulations and advanced analytics based studies on wild-type and hot-spot mutant A59G HRas
Source: PLoS One. 2020 Oct 16;15(10):e0234836. doi: 10.1371/journal.pone.0234836 (PMC7567374; doi:10.1371/journal.pone.0234836)

**Supplementary Information:**

**MGBSA based free energy analyses:**

The wild-type and mutant ensembles are thought to have distinct energetic trends, where the mutant conformations tend to be stuck in the active state due to large energetic barriers between the active and inactive states. In this regard, MMGBSA based energy based calculation was performed in the last 2000 ns for the wild-type and mutant trajectories namely: *simulationW* and *simulationM,* respectively. S1(a) Fig S1 and S1 (b) shows the trend of different energy components calculated for the two simulation trajectories. The coulombic energy values for the wild-type was -2000 KJ/mol and -1000 KJ/mol for the mutant trajectory, which showed a significant difference of 1000 KJ/mol between the two systems. Similarly, the polar solvation energy component also showed clear difference between the wild-type and mutant systems with energy values ranging around 2200 KJ/mol and 800 KJ/mol, for the respective systems. On the other hand, the van-der-waal component did not differ much for the two systems. Finally, the deltaG energy values showed a comparatively stable mutant conformation at -750 KJ/mol, as compared to the 0 to +-50 KJ/mol for the wild-type system, as depicted in Fig S1(c). Hence, the energy component could also be used as a distinguishing feature for the two systems for the feature-based PCA analysis (as discussed in the main article).

**MSM based analyses of MD simulation trajectories:**

Vamp scores were calculated before MSM based analysis was performed for the simulation trajectories. Fig S2 shows the VAMP score calculated for 3 different collective variables (CVs) or features namely: Dihedral angles (labelled as backbone torsions), minimum distance between 2 groups i.e. mindist (labelled as residue_mean) and distance between C-alpha atoms of the residue pair (labelled as CaDistance). The VAMP score calculation was performed on the control dataset i.e. the wild-type RAS MD simulation trajectory. The calculations were done at 3 different lag-time values i.e.: 0.5 ns, 1 ns and 2 ns respectively.

Fig S3 (a) and (b) shows the plot for estimation of appropriate value of the lag-time for the wild-type and mutant trajectories, respectively. As it is better to have a lag-time value sufficiently away from the gray area, because in this area it is assumed that the process under investigation has already decayed. Hence, a lag-time of 125 ns was chosen for both of the trajectories.

Further, the Chapman–Kolmogorov test (ck-test) was also performed to validate the selected lag-time value for the respective trajectories. Fig S4 (a) and 4 (b) shows the respective plots to demonstrate the confidence amongst the estimated and predicted lag-time values. Both the plots show that the chosen lag-time values are optimum for the respective systems.

**Supplementary figures and legends:**
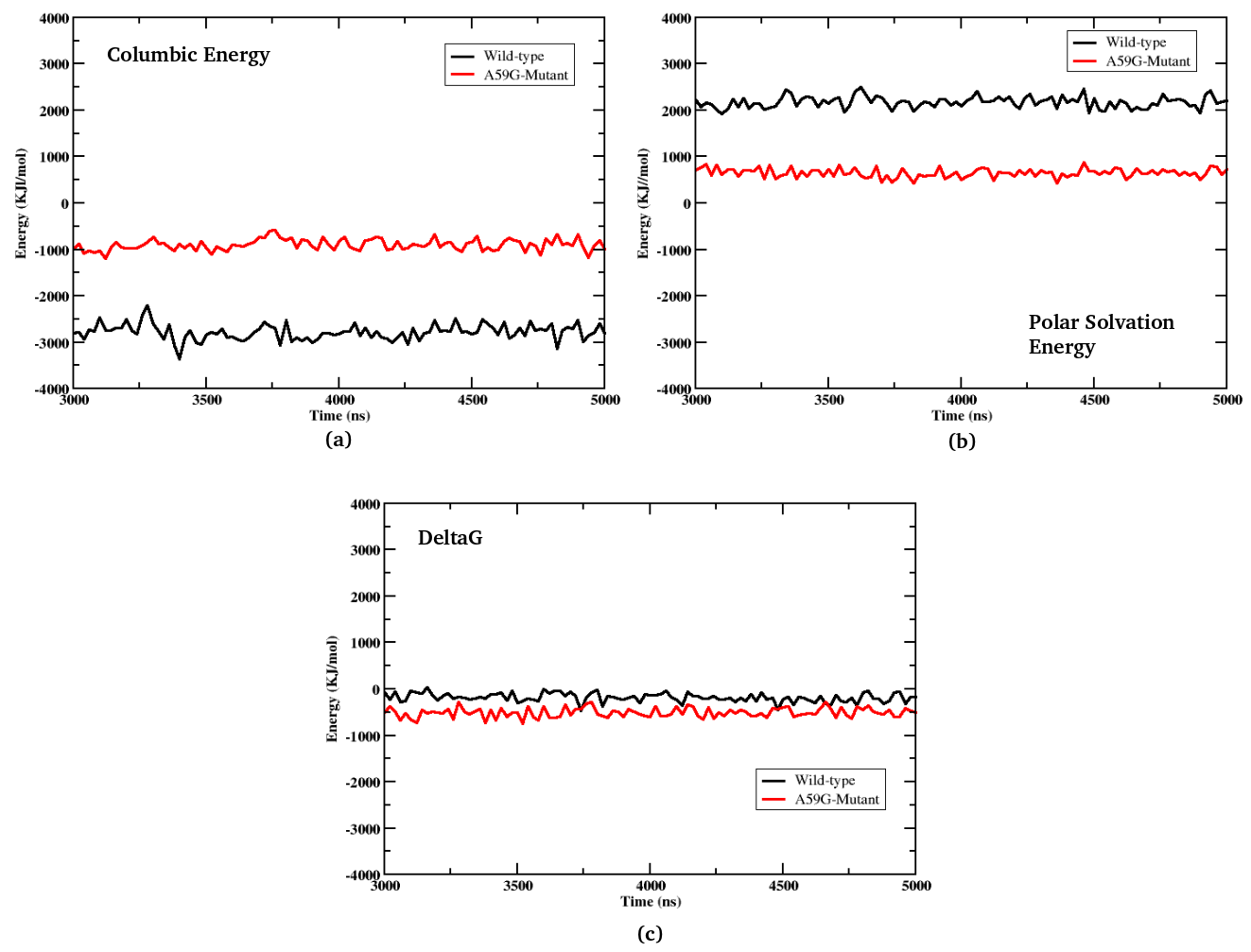


**Fig S1:** Figure showing the difference in energy components: (a) Columbic, (b) Polar solvation and (c) delta G, calculated for wild-type and mutant trajectories shown in black and red color representation, respectively.


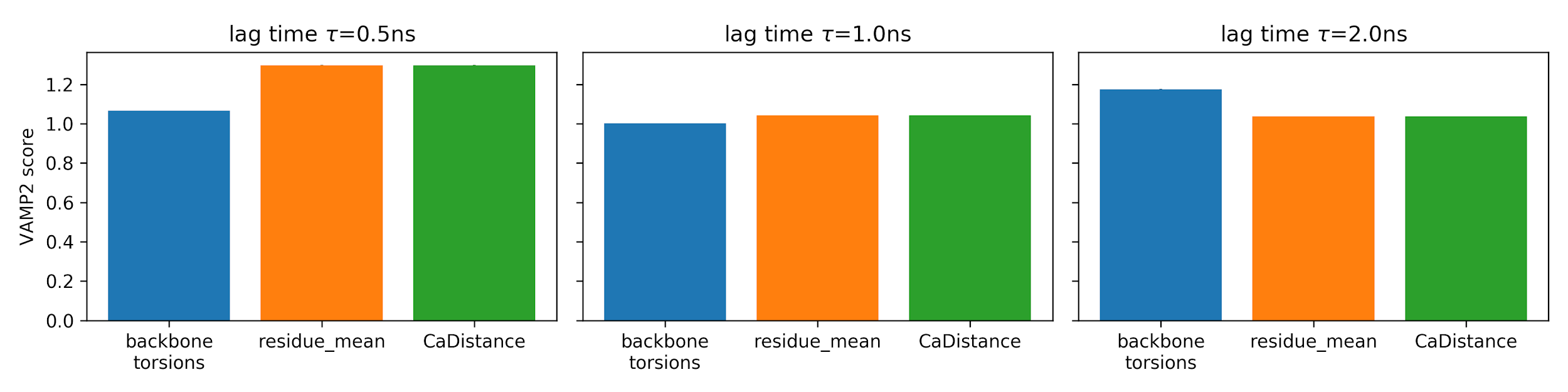


**Fig S2:** The graph showing vamp score calculation for 3 different CVs (dihedrals, residue mindist and Cadist) captured for 3 lag-time values: 0.5ns, 1ns and 2ns, respectively.

**Fig S3:** The figure demonstrates the relaxation time-scale against the individual lag-times, for wild-type and mutant MSM-based calculation. A lag-time of 125 ns (oval), was chosen for both of the trajectories.
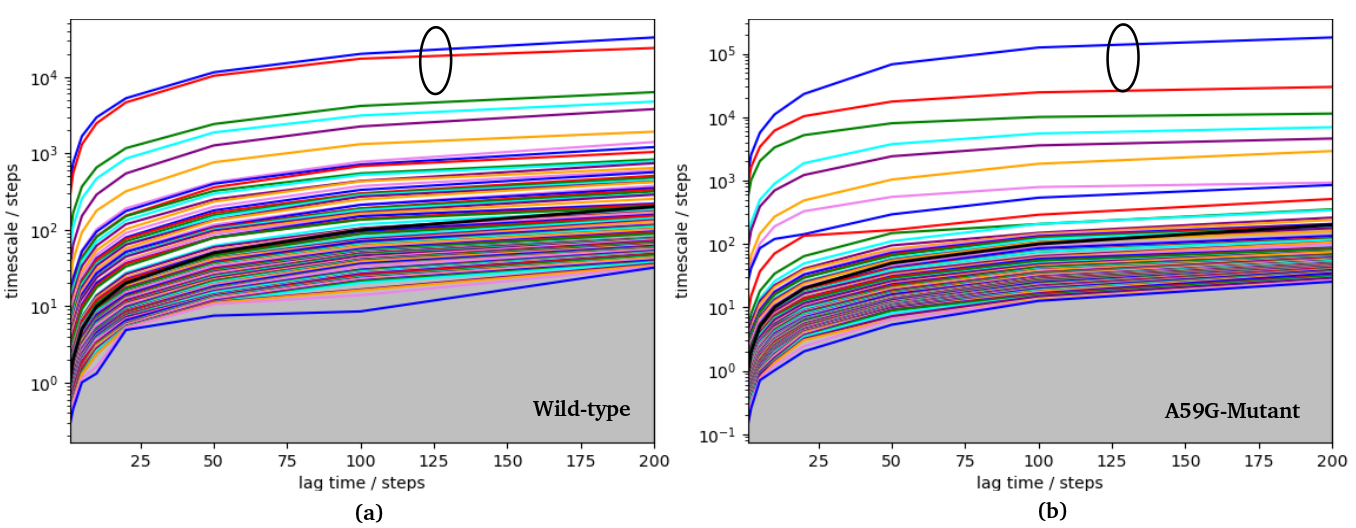


**Fig S4:** The plots depicts the output of Chapman-Kolpogorov test, accounting the correctness of the chosen-lag-time, for the (a) wild-type and (b) A59G-mutant systems, respectively.
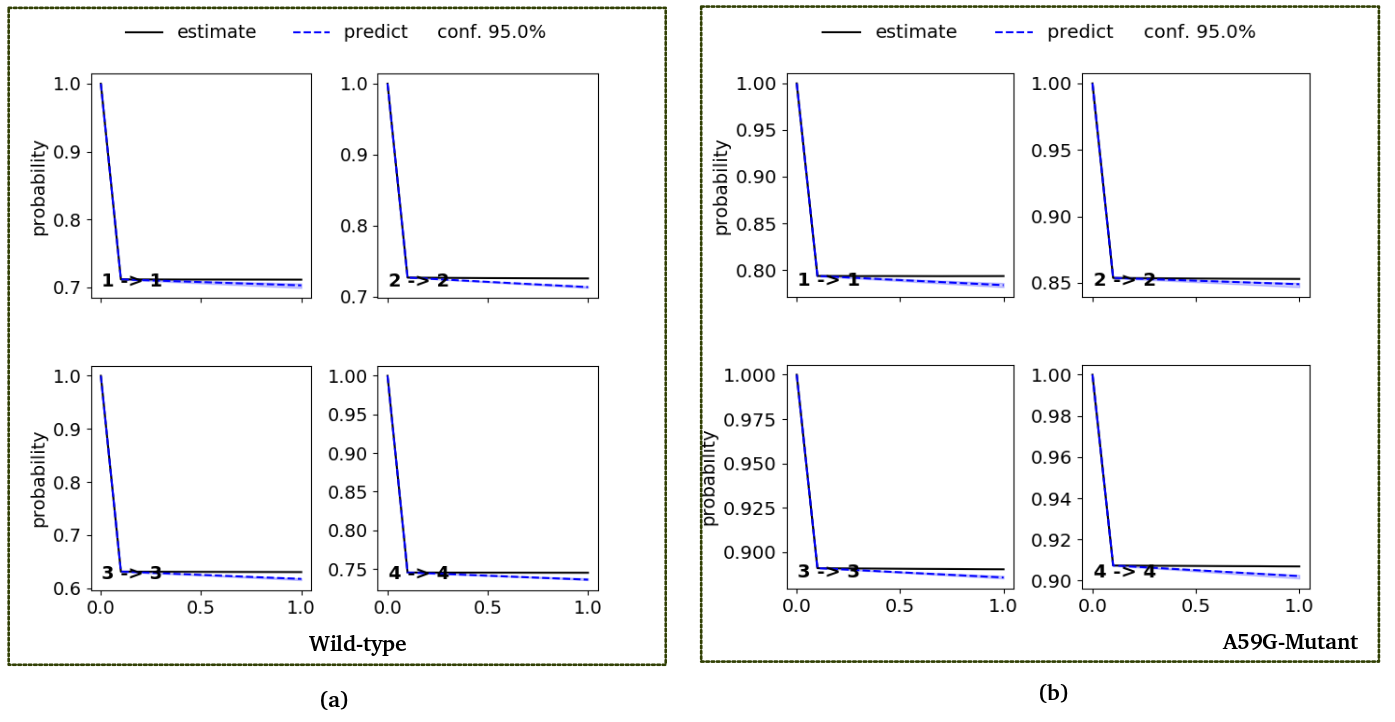

Supplement: S1 File — (DOCX) [file pone.0234836.s001.docx]
